# Genetic regulatory networks of soybean seed size, oil and protein contents

**DOI:** 10.3389/fpls.2023.1160418

**Published:** 2023-03-07

**Authors:** Zongbiao Duan, Qing Li, Hong Wang, Xuemei He, Min Zhang

**Affiliations:** ^1^ Hainan Yazhou Bay Seed Laboratory, Sanya, China; ^2^ State Key Laboratory of Plant Cell and Chromosome Engineering, Institute of Genetics and Developmental Biology, Chinese Academy of Sciences, Beijing, China; ^3^ State Key Laboratory of Rice Biology and Breeding, China National Rice Research Institute, Chinese Academy of Agricultural Sciences, Hangzhou, China

**Keywords:** soybean, seed size, oil, protein, QTL, functional genome

## Abstract

As a leading oilseed crop that supplies plant oil and protein for daily human life, increasing yield and improving nutritional quality (high oil or protein) are the top two fundamental goals of soybean breeding. Seed size is one of the most critical factors determining soybean yield. Seed size, oil and protein contents are complex quantitative traits governed by genetic and environmental factors during seed development. The composition and quantity of seed storage reserves directly affect seed size. In general, oil and protein make up almost 60% of the total storage of soybean seed. Therefore, soybean’s seed size, oil, or protein content are highly correlated agronomical traits. Increasing seed size helps increase soybean yield and probably improves seed quality. Similarly, rising oil and protein contents improves the soybean’s nutritional quality and will likely increase soybean yield. Due to the importance of these three seed traits in soybean breeding, extensive studies have been conducted on their underlying quantitative trait locus (QTLs) or genes and the dissection of their molecular regulatory pathways. This review summarized the progress in functional genome controlling soybean seed size, oil and protein contents in recent decades, and presented the challenges and prospects for developing high-yield soybean cultivars with high oil or protein content. In the end, we hope this review will be helpful to the improvement of soybean yield and quality in the future breeding process.

## 1 Introduction

**Figure 1:**
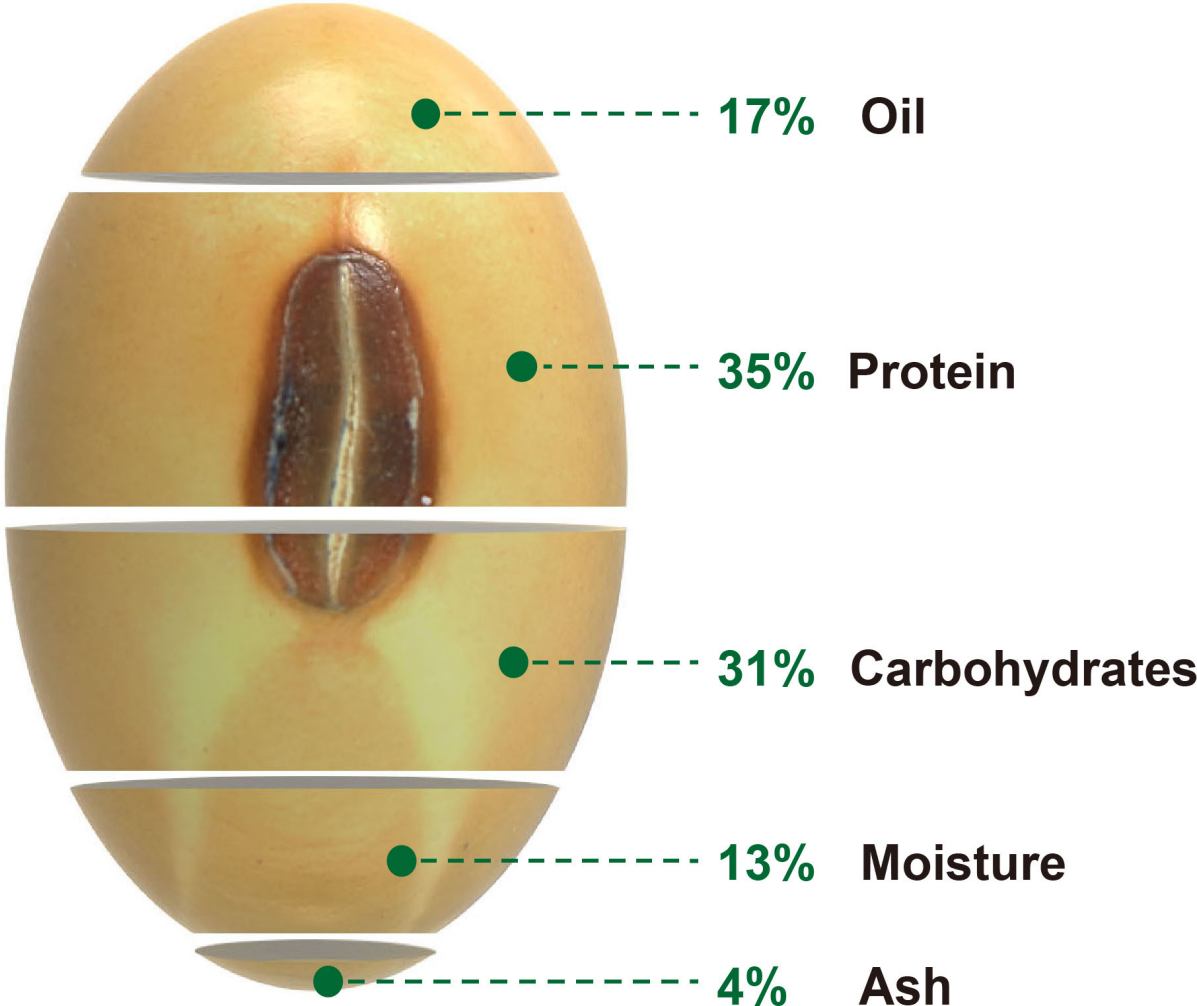
Composition of stored mature soybean seeds. The percentage value indicates the relative weight of the corresponding component in a seed (Liu, 1997).

Oil and protein are essential nutrients for humans and livestock, with almost 70% of cooking oil and half of feed protein coming from plants. Soybean (*Glycine max*) provides nearly 60% of global oilseed production and accounts for more than 25% of the protein consumption for food and animal feed worldwide, making it a leading commercial crop for vegetable oil and protein production (Wang et al., 2020b). The cultivated soybean was domesticated from wild soybean (*Glycine soja*) in central China about 5000 years ago and then spread around the world (Carter et al., 2004; Wilson, 2008). As a dominant oilseed and fodder crop, modern cultivated soybean seeds contain approximately 17% oil, 35% protein (including essential and non-essential amino acids), 31% carbohydrates (including soluble and insoluble carbohydrates), 13% moisture, and 4% ash (Liu, 1997) (**Figure 1**). The oil content of soybean seeds ranges from 8.3 to 27.9%, and protein concentration varies from 34.1 to 56.8% depending on the soybean varieties and cultivation conditions (Wilson, 2004). Soybean oil is generated and stored mainly as fatty acids (FAs), triacylglycerols (TAGs), and tocopherols (Liu et al., 2022). There are five central FAs presented in soybean seeds, including stearic acid (C18:0), oleic acid (C18:1), linoleic acid (C18:2), linolenic acid (C18:3), and palmitic acid (C16:0), whose composition directly determined the soybean oil quality. Soybean seed protein consists mainly of storage proteins such as glycinin (11S globulin) and conglycinin (7S globulin) (Liu et al., 2022).

Recent advances have shown that global crop yields need to be doubled by 2050 to keep up with the growing population and consumption (Godfray et al., 2010; Tilman et al., 2011), which means a 2.4% increase in crop production per year. However, soybean production seriously lags behind the projected demand, growing by an average of only 1.3% per year (Ray et al., 2013). Compared with staple crops, including rice, wheat, and maize, soybean yield is about one-third to one-half as much. Therefore, improving soybean yield is an essential and urgent task for soybean breeding. Increasing seed size is one of the crucial ways to boost soybean yield. Soybean seed size can be described using length (diameter parallel to the hilum), width (diameter from the hilum to the abaxial surface of seed), and thickness (diameter vertical to the hilum), and the composition and content of seed storage reserves directly determine it. Cultivated soybeans generally produce larger seeds with a higher oil level (Wang et al., 2020b). Wild soybeans have smaller seeds with lower oil content than cultivated soybeans. However, the seed protein content is not increased in the large-seed soybean cultivars (Wang et al., 2020b). Therefore, soybean improvement involves parallel increases in seed size, oil accumulation, and a possibly accompanying change in protein level.

For decades, increasing seed size, oil accumulation, and protein content have been the essential objectives of soybean breeding programs. The publication of the soybean reference genome (Williams 82) in 2010 has extensively promoted the development of soybean functional genomics (Zhang et al., 2022). Here, we review the advances in soybean functional genomics on seed size, oil accumulation, and protein content. In addition, we also discuss the challenges and prospects for developing high-yield soybean cultivars with high oil or protein content. As the biochemical synthesis of oils in the seed has been widely studied and well-reviewed (Bates et al., 2013; Xu and Shanklin, 2016; Song et al., 2017; Liu et al., 2022; Yang et al., 2022a), we will not repeat these comments here.

## 2 Genetic mapping associated with seed size, oil and protein contents

**Figure 2:**
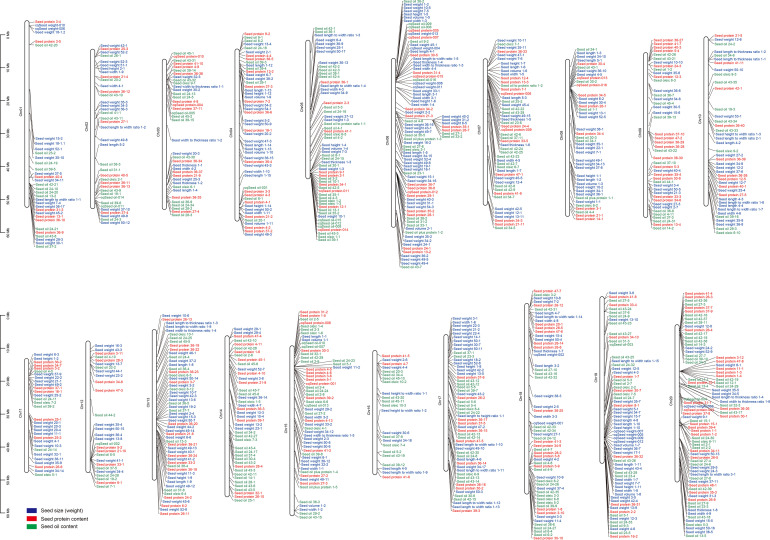
QTLs related to seed size (weight), oil accumulation, and protein content in soybean. These QTLs are derived from the SoyBase database (https://soybase.org/).

Seed size, oil and protein contents are complex traits controlled by genetic and environmental factors during seed development and maturation. Given their importance in soybean breeding, researchers have performed extensive linkage analysis to identify quantitative trait loci (QTL) associated with these three seed traits using various bi-parental derived populations, such as F2 population, recombinant inbred lines (RILs), chromosome segment substitution lines (CSSLs), and near-isogenic lines (NILs) (Han et al., 2012; Eskandari et al., 2013a; Eskandari et al., 2013b; Qi et al., 2014; Warrington et al., 2015; Wang et al., 2015a; Yang et al., 2019; Cui et al., 2020; Kumawat and Xu, 2021; Kumar et al., 2022; Luo et al., 2022; Yang et al., 2022b). So far, hundreds of QTLs related to seed size (including seed weight), oil accumulation, and protein content have been documented in the SoyBase Genome Database (http://www.soybase.org). For instance, there are 396 QTLs for seed size and weight (**Figure 2**; **Supplementary Table 1**), 333 QTLs for seed oil content (**Figure 2**; **Supplementary Table 2**), and 234 QTLs for seed protein content (**Figure 2**; **Supplementary Table 3**). Among these QTLs, some of the seed size, oil accumulation, and protein content-related QTLs shared overlapping regions, suggesting the presence of pleiotropic regulatory genes in these QTLs. However, due to the low-resolution and low-density molecular markers and limited population size, most QTLs were mapped in a large chromosome region, making these QTLs less effective in pinpointing the specific gene for crop improvement. At present, only a few genes involved in seed size, oil accumulation, and protein content have been isolated from QTL mappings, such as *GmPP2C-1* (Lu et al., 2017), *GmB1* (Zhang et al., 2018a), and *Glyma.20G85100* (also known as *GmSWEET39*) (Zhang et al., 2020; Fliege et al., 2022). In addition, two genes related to seed size/weight were identified through mutant-dependent map-based cloning or comparative genome hybridization (CGH) analysis, including *GmSSS1* (Zhu et al., 2022) and *GmKIX8-1* (Nguyen et al., 2021).

With the development of omics, genome-wide association study (GWAS) has become a powerful gene or QTL mapping approach for analyzing complicated agronomic traits in crops. Compared with conventional QTL mapping or linkage analysis, GWAS offers significant advantages: 1) GWAS does not need to build a mapping population. 2) GWAS population includes more natural variation than the bi-parental population. 3) GWAS can achieve higher mapping resolution due to high-density molecular markers and diverse historical recombination events (Wang et al., 2020a; Li et al., 2022b). Over the past decade, dozens of GWAS have been performed to identify QTLs or quantitative trait nucleotides (QTNs) involving seed size, lipid accumulation, and protein level in soybean (Hwang et al., 2014; Zhou et al., 2015; Zhang et al., 2016a; Yan et al., 2017; Zhang et al., 2018b; Lee et al., 2019; Zhao et al., 2019; He et al., 2021; Zhang et al., 2021; Hong et al., 2022). Based on this approach, *GmOLEO1* (Zhang et al., 2019b), *GmPDAT* (Liu et al., 2020a), *GmSWEET10a* (also known as *GmSWEET39*) (Miao et al., 2020; Wang et al., 2020b), and *GmST05* (Duan et al., 2022) have been identified and confirmed to relate to these seed traits, suggesting this way is more effective. Although GWAS has advantages in genetic mapping, the population structure and individual relationships are likely to produce false positive results in association analysis. Therefore, it is better to integrate linkage mapping and GWAS analysis for dissecting complex traits. Mixed analysis methods have successfully employed and mapped QTLs or QTNs associated with these seed traits in soybean (Cao et al., 2017; Zhang et al., 2019c), and further cloned *GmSWEET39* (Zhang et al., 2020), *GmGA3ox1* (Hu et al., 2022), *GmST1* (Li et al., 2022a), and *POWR1* (Goettel et al., 2022).

## 3 Regulatory genes of seed size

The seeds of higher plants consist of the embryo, endosperm, and seed coat, among which the embryo and endosperm are generated from the fertilized egg cell and central cell, respectively. In contrast, the seed coat is developed from the sporophytic integument. Therefore, seed size is determined by the integrated signals of maternal and zygotic tissues that influence the coordinated growth of the embryo, endosperm, and seed coat (Li et al., 2019). Several signaling pathways that maternal control seed size have been identified in *Arabidopsis* and rice, such as G-protein signaling, ubiquitin-proteasome signaling, mitogen-activated protein kinase (MAPK) signaling, phytohormone signaling, and some transcriptional regulators. Meanwhile, the HAIKU (IKU) pathway and some phytohormones partially regulate the zygotic tissues’ growth (Li et al., 2019). However, compared with *Arabidopsis* and rice, the molecular networks regulating seed size in soybean are still lagging behind.

**Figure 3:**
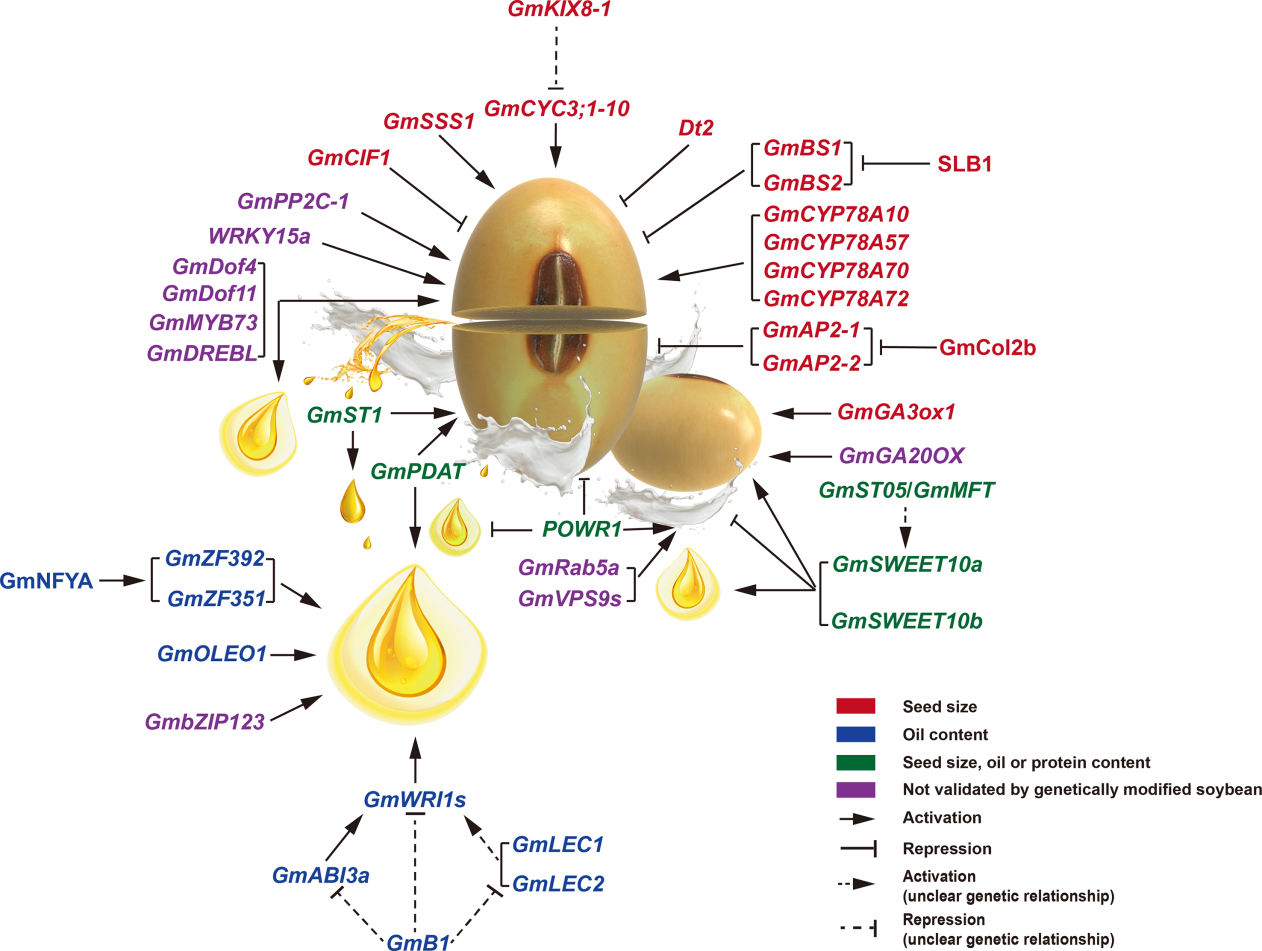
Genetic regulatory network of seed size (weight), oil accumulation, and protein content in soybean. The genes or proteins involving seed size (weight) and oil content are shown in red and blue fonts, respectively. The pleiotropic regulators for seed size (weight), oil accumulation, or protein content are indicated in green fonts. The regulatory genes, whose function has been validated only in *Arabidopsis* but not soybean, are shown in purple fonts.

As critical regulatory components of gene expression, several transcriptional factors (TFs) involved in seed size have been identified in soybean (**Figure 3**; **Table 1**). *BIG SEEDS1* (*BS1*) belongs to a group II member of the TIFY TF family. It plays a vital role in controlling the size of seeds, pods, and leaves *via* a regulatory module that targets cell proliferation in the model legume of *Medicago truncatula* (Ge et al., 2016). Down-regulation of *BS1* orthologous genes (*GmBS1* and *GmBS2*) in soybean resulted in increased seed size and amino acid content. *SLB1* encodes an F-box protein that forms part of the SKP1/Cullin/F-box E3 ubiquitin ligase complex. Biochemical and genetic analyses showed that SLB1 interacts with BS1 to control lateral branching and organ growth by regulating BS1 protein stability in *Medicago truncatula*. In addition, overexpression of *SLB1* resulted in increased leaf and seed size in both *Medicago truncatula* and soybean, suggesting the functional conservation of *SLB1* (Yin et al., 2020). Plant WRKY TFs are involved in many biological processes, such as embryogenesis and seed development (Luo et al., 2005). The *WRKY15a* was differentially expressed during pod development between cultivated and wild soybeans. Four haplotypes (H1-H4) were present in *WRKY15a*, which varied in the CT-core microsatellite locus at the 5’-untranslated region (5’-UTR) of *WRKY15a*. The H1 haplotype with six CT-repeats was the only allele in cultivated soybeans, whereas the H3 haplotype with five CT-repeats was the primary allele in wild soybeans. The seed weight with haplotype H1 was heavier than that of wild soybeans harboring haplotypes H2, H3, and H4, and the seed weight was positively correlated with *WRKY15a* expression, indicating a positive effect of *WRKY15a* on seed size (Gu et al., 2017). *Dt2*, encoding a MADS-box TF, plays an essential role in controlling multiple agronomic traits, such as flowering time, stem growth habit, and plant height (Ping et al., 2014; Zhang et al., 2019a). A recent report has shown that *Dt2* also determines shoot branching and seed size (Liang et al., 2022). *Dt2* knockout lines performed multiple yield-related trait changes, such as the increased seed length and width, heavier seed weight, and higher grain weight per plant, thereby resulting in obviously improved yield per plot. In contrast, the *Dt2* overexpression lines exhibited decreased seed length and width.

**Table 1:** Representative genes related to seed size, oil accumulation, and protein content in soybean.

Gene	Accession No. (Wm82.a2.v1)	Functional annotation	Controlling trait	References
*GmCYP78A10*	Glyma.05G019200	cytochrome P450 CYP2 subfamily	seed size/weight	Wang et al., 2015b
*GmCYP78A57*	Glyma.02G119600	cytochrome P450 CYP2 subfamily/homolog of AtCYP78A6/KLU	seed size/weight	Zhao et al., 2016
*GmCYP78A70*	Glyma.01G061100	cytochrome P450 CYP2 subfamily/homolog of AtCYP78A7/KLU	seed size/weight	Zhao et al., 2016
*GmCYP78A72*	Glyma.19G240800	cytochrome P450 CYP2 subfamily/homolog of AtCYP78A5/KLU	seed size/weight	Zhao et al., 2016
*GA20OX*	Glyma.07G081700	gibberellin 20 oxidase	seed size/weight	Lu et al., 2016
*GmBS1*	Glyma.10G244400	a group II member of the TIFY family of transcription regulators	seed size/weight	Ge et al., 2016
*GmBS2*	Glyma.20G150000	a group II member of the TIFY family of transcription regulators	seed size/weight	Ge et al., 2016
*WRKY15a*	Glyma.05G096500	WRKY transcription factor	seed size/weight	Gu et al., 2017
*GmPP2C-1*	Glyma.17G221100	a putative phosphatase 2C protein	seed size/weight	Lu et al., 2017
*GmCIF1*	Glyma.17G036300	cell-wall inhibitor of beta-fructosidase	seed size/weight	Tang et al., 2017
*GmKIX8-1*	Glyma.17G112800	unknow protein	seed size/weight	Nguyen et al., 2021
*GmSSS1*	Glyma.19G196000	SPINDLY homolog protein	seed size/weight	Zhu et al., 2022
*GmGA3ox1*	Glyma.07G033800	gibberellin 3β-hydroxylase	seed size/weight	Hu et al., 2022
*Dt2*	Glyma.18G273600	MADS-box transcription factor	seed size/weight	Liang et al., 2022
*GmCOL2b*	Glyma.19G039000	B-box type zinc finger protein with CCT domain	seed size/weight	Yu et al., 2023
*GmAP2-1*	Glyma.01g188400	AP2 transcription factor	seed size/weight	Yu et al., 2023
*GmAP2-2*	Glyma.05g091200	AP2 transcription factor	seed size/weight	Yu et al., 2023
*GmbZIP123*	Glyma.06G010200	bZIP transcription factor	oil content	Song et al., 2013
*GmNFYA*	Glyma.02G303800	a domain of the nuclear transcription factor Y	oil content	Lu et al., 2016
*GmLEC1*	Glyma.07G268100	nuclear transcription factor Y subunit B	oil content	Pelletier et al., 2017
*GmLEC2a*	Glyma.20G035800	AP2/B3-like transcriptional factor	oil content	Manan et al., 2017
*GmLEC2b*	Glyma.20G035700	AP2/B3-like transcriptional factor	oil content	Manan et al., 2017
*GmZF351*	Glyma.06G290100	zinc finger C-x8-C-x5-C-x3-H type family protein	oil content	Li et al., 2017
*GmWRI1a*	Glyma.15G221600	ethylene-responsive transcription factor WRI1a	oil content	Chen et al., 2018
*GmB1*	Glyma.13G241700	a transmembrane transporter-like protein	oil content	Zhang et al., 2018a
*GmOLEO1*	Glyma.20G196600	oleosin-encoding gene	oil content	Zhang et al., 2019b
*GmPDAT*	Glyma.13G108100	regulator of chromosome condensation	oil content	Liu et al., 2020a
*GmWRI1b*	Glyma.08G227700	ethylene-responsive transcription factor WRI1b	oil content	Guo et al., 2020
*GmZF392*	Glyma.12G205700	zinc finger C-x8-C-x5-C-x3-H type family protein	oil content	Lu et al., 2021
*Rab5a2*	Glyma.13G153000	a small GTPase	protein content	Wei et al., 2020
	Glyma.18G045000	a small GTPase	protein content	
*GmDof4*	Glyma.17G081800	Dof zinc finger protein	seed size/weight and oil content	Wang et al., 2007
*GmDof11*	Glyma.13G329000	Dof zinc finger protein	seed size/weight and oil content	Wang et al., 2007
*GmMYB73*	Glyma.06G303100	MYB transcription factor MYB73	seed size/weight and oil content	Liu et al., 2014
*GmDREBL*	Glyma.12G103100	DREB-type transcription factors	seed size/weight and oil content	Zhang et al., 2016b
*GmST1*	Glyma.08G109100	UDP-D-glucuronate 4-epimerase	seed size/weight and oil content	Li et al., 2022a
*GmSWEET10a*	Glyma.15G049200	sugar efflux transporter SWEET39	seed size/weight, oil and protein contents	Wang et al., 2020b; Miao et al., 2020
*GmSWEET10b*	Glyma.08G183500	sugar efflux transporter SWEET24	seed size/weight, oil and protein contents	Wang et al., 2020b
*GmST05*	Glyma.05G244100	phosphatidylethanolamine-binding protein	seed size/weight, oil and protein contents	Duan et al., 2022
*POWR1*	Glyma.20G085100	CCT domain protein	seed size/weight, oil and protein contents	Fliege et al., 2022; Goettel et al., 2022

Some genes that encode various enzymes have also been shown to affect soybean seed size (**Figure 3**; **Table 1**). A phosphatase 2C-1 (*GmPP2C-1*) gene from wild soybean helps to increase seed weight or size by improving integument cell size and activating a subset of seed trait-related genes (Lu et al., 2017). In addition, GmPP2C-1 facilitates the accumulation of dephosphorylated GmBZR1 protein, which act as the key transcription factor in BR signaling. Furthermore, overexpression of *GmBZR1* can improve seed size or weight in transgenic *Arabidopsis*. Cell wall invertase (CWI) plays a vital role in sugar signaling and metabolism, affecting the source–sink interaction and seed development (Tang et al., 2017). *GmCIF1* encodes a cell wall invertase inhibitor, and suppression of *GmCIF1* gene expression exhibited increased CWI activities and larger seed size while with more accumulations of protein, hexoses, and starch in soybean seeds. *GmSSS1* encodes a putative O-GlcNAc transferase in soybean. Knockout *GmSSS1* resulted in tiny seeds, whereas overexpressing *GmSSS1* produced large seeds (Zhu et al., 2022). Modulating *GmSSS1* could positively affect cell division and expansion in transgenic plants. GmGA3ox1, a gibberellin (GA) 3β-hydroxylase in soybean, is the critical enzyme in the GA biosynthesis pathway. Knockout of *GmGA3ox1* resulted in reduced GA biosynthesis while enhanced photosynthesis (Hu et al., 2022). *GmGA3ox1* knockout plants displayed decreased seed weight and length, but improved seed production by increasing branch, pod, and seed numbers. In contrast, overexpression of *GmGA3ox1* increased seed weight and length in transgenic soybeans. Similarly, overexpression of *GA20OX*, encoding a gibberellin 20 oxidase in a rate-limiting step of GA biosynthesis, enhanced the seed size/weight of transgenic *Arabidopsis* plants (Lu et al., 2016).

Besides the above genes, some homologous soybean genes known to regulate seed size in *Arabidopsis* have also been shown to control soybean seed size (**Figure 3**; **Table 1**). For example, several *P450/CYP78A* family members are suggested for controlling seed size in *Arabidopsis* (Wang et al., 2008; Fang et al., 2012). The *P450/CYP78A* orthologs in soybean, such as *GmCYP78A10*, *GmCYP78A57*, *GmCYP78A70*, and *GmCYP78A72*, exhibited conserved function to improve seed size or weight (Wang et al., 2015b; Zhao et al., 2016; Du et al., 2017), but the underlying mechanism how they function remains largely elusive. A PPD/KIX/TPL repressor complex consisting of PPD2, KIX8/9, and TPL proteins was shown to affect organ size by modulating meristem proliferation in *Arabidopsis* (Baekelandt et al., 2018). *GmKIX8-1*, a soybean *AtKIX8* ortholog, is also involved in controlling cell proliferation and organ size. Due to increased *CYCLIN D3;1-10* expression and cell proliferation, the *GmKIX8-1* loss-of-function mutants displayed an apparent increase in the size of leaves and seeds (Nguyen et al., 2021). Very recently, in both *Arabidopsis* and soybean, a crucial regulatory cascade involving CO (the central regulator of the photoperiodic pathway) and AP2 (specification of floral meristem identity) was demonstrated to mediate the photoperiod-regulated seed size in a maternal-dependent manner (Yu et al., 2023). GmCOL2b (a soybean CO homolog) positively promoted seed size under short days by directly inhibiting the expression of *GmAP2-1* and *GmAP2-2*.

## 4 Regulatory genes of seed oil

Seed storage reserves, including oil, protein, and starch, are filled during seed development and maturation. Understanding the storage substance loading into the seeds thus is crucial to improving crop yield and nutritional quality. In the past decades, extensive efforts have been made toward the dissection of molecular pathways for accumulating seed storage reserves, particularly in *Arabidopsis*. TFs, such as LEC1, LEC2, ABI3, FUS3, and WRI1, and other activators or repressors for storage reserves accumulation during seed development, have been identified in plants (Yang et al., 2022a). However, more details and mechanisms have yet to be clarified, especially for essential crops such as soybean (**Figure 3**; **Table 1**).

LEC1 is an atypical TF subunit (NF-YB) that interacts with NF-YA and NF-YC subunits to form an NF-Y TF complex. It is central to controlling seed development, such as embryo morphogenesis, endosperm development, and storage reserve accumulation (Jo et al., 2019). In *Arabidopsis*, the *lec1* null mutants displayed striking defects in embryos and severely restricted protein and lipid accumulation in seeds (Meinke et al., 1994; West et al., 1994). Furthermore, over-expression of *LEC1* induced the activation of genes related to the accumulation of storage proteins and lipids, resulting in increased contents of lipids and FAs in the transgenic *Arabidopsis* (Kagaya et al., 2005). In soybean, *GmLEC1* (*GmLEC1a* or *GmLEC1b*) transcriptionally regulates the genes involved in distinct cellular processes during seed development and activates seed FAs biosynthesis (Pelletier et al., 2017; Zhang et al., 2017). Further research revealed that GmLEC1 acts in combination with TFs such as GmAREB3, GmbZIP67, and GmABI3 to regulate soybean seed development (Jo et al., 2020).

LEC1 interacts physically with LEC2, a B3 DNA binding domain TF, which has a crucial regulatory role in seed development and in controlling seed protein and oil levels in *Arabidopsis* (Santos-Mendoza et al., 2008; Angeles-Núñez and Tiessen, 2011; Kim et al., 2015; Jo et al., 2019). The loss-of-function *lec2* mutant seeds showed a 30% and 15% decline in oil and protein, respectively, but accumulated more starch and sucrose than wild-type seeds (Angeles-Núñez and Tiessen, 2011). In contrast, in both transgenic *Arabidopsis* and tobacco plants, *AtLEC2* inducible expression increased storage oil accumulation, such as TAGs and FAs (Mendoza et al., 2005; Andrianov et al., 2010; Kim et al., 2015). In soybean, *GmLEC2* regulates a subset of genes involving the metabolism of seed storage reserves (Manan et al., 2017). Compared with the control seeds, the TAGs and long-chain FAs contents of GmLEC2a *over-expression* transgenic Arabidopsis seeds increased by 34% and 4%, respectively.

In the transcriptional network of seed oil accumulation in *Arabidopsis*, LEC1 and LEC2 synergistically promote *WRI1* expression, an AP2 TF gene responsible for the transcriptional regulation of oil biosynthesis, and this regulatory mechanism is conserved in other plant species, for instance, soybean and maize (Baud et al., 2007; Mu et al., 2008; Shen et al., 2010; Manan et al., 2017; Pelletier et al., 2017; Yang et al., 2022a). Its two soybean orthologs, *GmWRI1a* and *GmWRI1b*, play a central role in seed oil accumulation. Over-expression of *GmWRI1a* or *GmWRI1b* significantly increased total oil and FAs contents and changed FAs composition in the seed, whereas *GmWRI1* knockdown hairy roots interfered with lipid biosynthesis (Chen et al., 2018; Chen et al., 2020; Guo et al., 2020; Wang et al., 2022).

GmZF392, a seed-specific tandem CCCH zinc finger (TZF) protein, promotes seed oil accumulation by targeting a bipartite cis-element with TA- and TG-rich sequences in the promoter regions, thereby activating downstream gene expression involving in the lipid biosynthesis (Lu et al., 2021). GmZF392 interacts physically with GmZF351, another activator of lipid accumulation, to additive/synergistic increase the expression of downstream lipid biosynthesis genes (Li et al., 2017; Lu et al., 2021). And both *GmZF392* and *GmZF351* are positively regulated by GmNFYA, a TF correlated with oil content (Lu et al., 2016; Lu et al., 2021). In addition, *GmZF392* and *GmZF35*1 are also direct targets of GmLEC1 (Pelletier et al., 2017). More importantly, *GmZF392* and *GmZF35*1 were selected by domestication from wild soybeans to cultivated soybeans.

In addition to the above TFs forming the regulatory module, some functional genes were also involved in regulating seed oil content in soybean (**Figure 3**; **Table 1**). Overexpression of a bZIP TF gene (*GmbZIP123*) enhances lipid accumulation in transgenic *Arabidopsis* seeds through modulating sugar transport (Song et al., 2013). *GmB1*, encoding a transporter-like transmembrane protein for the biosynthesis of the bloom in pod endocarp, not only controls seed coat bloom in wild soybeans but also affects oil content in cultivated soybeans (Zhang et al., 2018a). *GmOLEO1*, a strong artificial-selected oleosin protein-encoding gene, conduces to the improvement in seed oil content during soybean domestication by affecting TAGs metabolism (Zhang et al., 2019b).

## 5 Regulatory genes of seed protein

Compared with seed size and oil content, only a few genes controlling seed protein or amino acid content have been functionally identified (**Figure 3**; **Table 1**) (Krishnan and Jez, 2018). The small GTPase GmRab5a and its guanine exchange factors GmVPS9s are shown to function in the storage protein post-Golgi trafficking in soybean (Wei et al., 2020). Transient over-expression of the dominant negative variant of *GmRab5a*, or RNAi of either *GmRab5a* or *GmVPS9s*, obviously reduced the transport of the cargo marker, which used to reflect storage protein trafficking to protein storage vacuoles in soybean cotyledon cells. In addition, several genes, including *POWR1*, *GmSWEET10a*, *GmSWEET10b*, and *GmST05*, pleiotropically regulate seed protein, oil content, and seed size (Wang et al., 2020b; Duan et al., 2022; Goettel et al., 2022), which are detailed discussion in the next section.

## 6 Pleiotropic regulatory genes of seed size, oil and protein contents

Seed size, oil accumulation, and protein content in soybean are highly correlated agronomical traits. However, the selection and underlying molecular basis of these seed-correlated traits during soybean domestication are poorly understood, which is one of the obstacles to soybean yield and quality improvement. So far, several pleiotropic regulatory genes controlling seed size, oil accumulation, and protein content have been cloned and functionally identified in soybean (**Figure 3**; **Table 1**).

For instance, the ectopic expression of *GmDof4*, *GmDof11*, *GmMYB73*, and *GmDREBL* enhanced both seed size/weight and oil accumulation in transgenic *Arabidopsis* seeds (Wang et al., 2007; Liu et al., 2014; Zhang et al., 2016b). *GmPDAT*, a phospholipid diacylglycerol acyltransferase encoding gene, was expressed higher in large-seed and high-oil soybean accessions than in small-seed and low-oil accessions. Over-expression of *GmPDAT* improved seed size and oil level, whereas *GmPDAT* RNAi plants had reduced seed size and oil accumulation (Liu et al., 2020a). *GmST1* encodes a UDP-D-glucuronate 4-epimerase that positively regulates seed size and oil content by modulating pectin biosynthesis and glycolysis pathways, and underwent selection during soybean domestication (Li et al., 2022a).

The sugar transporter *SWEET* family members play critical roles in seed development (Chen et al., 2015; Wang et al., 2019). A pair of *SWEET* paralogs in soybean, *GmSWEET10a* and *GmSWEET10b*, underwent the stepwise selection that synchronously changed seed size, oil accumulation, and protein level during soybean domestication, by regulating sugar sorting from seed coat to embryo (Zhang et al., 2020; Wang et al., 2020b). Compared with wild-type plants, *GmSWEET10a* or *GmSWEET10b* over-expression soybeans displayed significantly increased seed size and higher oil accumulation but decreased protein level, while their knockout plants had reduced seed size and oil content but increased protein level (Wang et al., 2020b). Very recently, a phosphatidylethanolamine-binding protein (PEBP) family member, *GmST05* (also known as *GmMFT*), has been shown to positively regulate seed size and altered oil and protein levels, likely by affecting *GmSWEET10a* transcription (Li et al., 2014; Duan et al., 2022). In addition, a CCT-domain gene, *POWR1*, is domesticated and pleiotropically regulates seed quality and yield in soybean, possibly by regulating lipid metabolism and nutrient transport (Goettel et al., 2022). A transposable element (TE) insertion in the CCT-domain of *POWR1* resulted in increased seed weight and oil content but decreased protein content. In contrast, over-expression of *POWR1* exhibited improved protein content and declined seed weight and oil accumulation in transgenic plants.

## 7 Challenges and perspectives

Seed size, oil and protein contents are complex quantitative traits governed by multiple genes. Although linkage mapping and GWAS analysis have identified numerous QTLs controlling seed size, oil accumulation, and protein content in soybean, only a few genes have been isolated and functionally validated. One fundamental reason for this phenomenon is that these researchers usually use only one or two approaches, making it hard to pinpoint the target underlying these seed traits. The other key obstacle is the lack of a fast and efficient soybean genetic transformation system for different soybean genotypes, such as *Agrobacterium*-mediated cotyledonary node soybean transformation, which has been widely used in recently years. The slow and inefficient genetic transformation system makes it more challenging to identify and verify the function of soybean genes (Zhang et al., 2022). That’s why, in some studies, especially those prior to 2015, functional validation was done in *Arabidopsis* instead of soybean.

With the rapid progress of omics research and the reduction of testing cost, more and more soybean omics data were produced, such as the re-sequencing genome, transcriptome, metabolome, proteome, epigenome, pan-genome, and 3D genome (Ohyanagi et al., 2012; Lin et al., 2014; Shen et al., 2014; Zhou et al., 2015; Liu et al., 2016; Fang et al., 2017; Shen et al., 2018a; Shen et al., 2018b; Liu et al., 2020b; Silva et al., 2021; Ni et al., 2023). These released omics resources will extensively promote the research of soybean functional genomics. Currently, like GWAS, TWAS (transcriptome-wide association study), EWAS (epigenome-wide association study), and PWAS (proteome-wide association study), as well as multi-omics data association studies, such as eGWAS (gene expression-based genome-wide association study) and mGWAS (metabolome-based genome-wide association study) have been successfully developed and applied (Shen et al., 2022). Integration of multiple omics approaches will provide more clues and help narrow the target range underlying these seed traits. However, utilizing these vast omics data that exist in various forms is a considerable challenge. Thus, mathematical methods, like meta-analysis, are expected to address such trouble. Moreover, artificial intelligence (AI) technology or machine learning approach can make mining big data more efficient, for instance, omics data processing, protein structure construction, and pan-omics data integration (Baek et al., 2021; Jumper et al., 2021; Reel et al., 2021).

CRISPR/Cas-based genome editing technology that enables precise modification of genomes to obtain predictable and desired traits has been successfully applied to gene function research and crop germplasm creation. Compared with other crops, such as rice, the soybean genome-editing process is primarily in its infancy; however, successful stories have demonstrated the feasibility of gene editing in soybean (Cai et al., 2018; Bai et al., 2020; Wang et al., 2020b; Nguyen et al., 2021; Bai et al., 2022; Duan et al., 2022; Hu et al., 2022; Liang et al., 2022; Li et al., 2022a). In the future, the improved soybean transformation and more applications of single - or multi-gene ‘base editing’ will greatly facilitate functional research in soybean, ultimately allowing us to decode these complex seed traits and identify critical genes underlying seed size, oil and protein contents.

The ultimate goal of soybean breeding is to cultivate high-yield and high-quality soybean. So far, crop breeding has developed from artificial selection (stage 1.0) and hybrid breeding (stage 2.0) to molecular breeding (stage 3.0). However, to solve the crisis of food shortage caused by the growing population, intelligent breeding (stage 4.0) that can quickly aggregate excellent alleles through precise design is coming (Shen et al., 2022). In previous breeding stages, breeders usually have to stack desirable traits into a single line to create a super variety, which is a huge task. In breeding stage 4.0, optimal and precise design to rapidly pyramid multiple elite alleles with desirable seed traits will facilitate yield, oil, and protein content improvement in soybean.

## Author contributions

QL and MZ designed and supervised the study. ZD, QL, and HW drafted the manuscript. XH participated in the production of the article pictures. ZD and QL responded to review comments. All authors contributed to the article and approved the submitted version.
